# Bilateral simultaneous laparoscopic clipless adrenal-sparing surgery for pheochromocytoma in a pediatric patient: A case report

**DOI:** 10.1016/j.eucr.2025.103072

**Published:** 2025-05-17

**Authors:** Nasser Simforoosh, Javad Nikbakht, Ahmad Eghbali, Nooshin Ahmadi, Mohammad Sajjad Zabihi, Saeedeh Sarhadi, Mehdi Dadpour

**Affiliations:** a Shahid Labbafinejad Medical Center, The Center of Excellence in Urology, Urology and Nephrology Research Center, Research Institute for Urology and Nephrology, Shahid Beheshti University of Medical Sciences, Tehran, Iran; b Anesthesiology Research Center, Mofid Children Hospital, Shahid Beheshti University of Medical Sciences, Tehran, Iran; c Research Institute for Endocrine Sciences, Shahid Beheshti University of Medical Sciences, Tehran, Iran; d Health Promotion Research Center, Zahedan University of Medical Sciences, Zahedan, Iran

**Keywords:** Bilateral, Adrenalectomy, Pheochromocytoma, Laparoscopy, Pediatrics

## Abstract

Herein, we describe an 11-year-old male with a known history of Von Hippel-Lindau disease. He presented with malignant hypertension and bilateral adrenal masses measuring approximately 5cm and 3cm on the right and left sides, respectively. Pheochromocytoma was confirmed through imaging and lab data. The patient successfully underwent bilateral simultaneous laparoscopic clipless adrenal-sparing surgery. At the six-month follow-up, he remained asymptomatic and normotensive, normal laboratory tests or imaging. Additionally, there were no signs of adrenal insufficiency, eliminating the need for corticosteroid or mineralocorticoid replacement therapy. Laparoscopic bilateral synchronous adrenal-sparing surgery represents a significant advancement in the management of pediatric bilateral pheochromocytoma.

## 1 Introduction

Bilateral adrenal pheochromocytoma, often associated with hereditary conditions such as multiple endocrine neoplasia type 2 and Von Hippel-Lindau disease (VHL), poses significant clinical challenges due to its potential to cause severe complications, including catecholamine-induced hypertension and cardiovascular risks.^1^^,^^2^ These tumors are marked by excessive catecholamine secretion, leading to symptoms like hypertension, tachycardia, anxiety, and blurred vision. Early diagnosis is critical to prevent substantial morbidity and mortality.^3^

Laparoscopic intervention is the mainstay treatment, with total adrenalectomy traditionally favored for bilateral tumors. However, this approach can result in adrenal insufficiency, necessitating lifelong hormone replacement therapy and its associated complications.^4^ Recently, laparoscopic partial adrenalectomy and adrenal-sparing surgeries have gained attention for preserving adrenal function while effectively removing tumor tissue. Studies highlight comparable recurrence and complication rates between partial and total adrenalectomy, making adrenal-sparing techniques increasingly appealing.^5^, ^6^, ^7^

Given the rarity of bilateral pheochromocytoma in children, data guiding surgical management are limited.^7^^,^^8^ Partial adrenalectomy offers a promising approach to mitigate adrenal insufficiency-related risks. This article reports our experience with simultaneous bilateral laparoscopic clipless adrenal-sparing surgery in a pediatric patient with pheochromocytoma, showcasing its benefits in preserving adrenal function and avoiding the need for postoperative hormone replacement.

## 2 Case report

**History and characteristics:** An 11-year-old boy with Von Hippel-Lindau (VHL) syndrome presented with a two-month history of headaches and blurred vision. His father had a history of adrenal mass removal for pheochromocytoma. Examination revealed severe hypertension (240/120 mmHg). CT scan identified a 52 × 45 mm right adrenal mass and a smaller 29 × 20 mm lesion on the left (Fig. 1). Laboratory tests showed elevated 24-h urine metanephrine and normetanephrine levels, confirming the diagnosis of bilateral pheochromocytoma.

**1):**
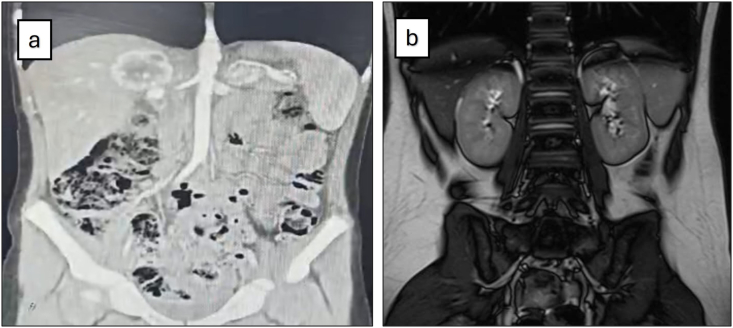
(a) CT scan revealed a hyperechoic mass measuring 52 × 45 mm in the anatomical position of the right adrenal gland and a similar but smaller solid lesion measuring 29 × 20 mm in the left adrenal gland. (b) Follow up MRI showed no residual masses.

**Pre-op hospitalization:** The patient was diagnosed with bilateral pheochromocytoma and was treated for hypertension in preparation for surgery. An endocrine consultation was obtained, and phenoxybenzamine was initiated at a dose of 10 mg every 8 hours that gradually increased to a maximum of 100 mg daily in the last two days before operation. The patient's blood pressure stabilized during hospitalization, and he was scheduled for surgery. On the day of the operation, the patient developed severe hypertension (systolic pressure of 220 mmHg) following anesthesia induction, prompting cancellation of the procedure. After further endocrine consultation, the dose of phenoxybenzamine started at 50 mg daily and gradually increased to 120 mg daily in the last two days before the operation. The patient was then rescheduled for surgery without any problems of high blood pressure.

**Procedure:** The surgery was performed using a laparoscopic transperitoneal approach. Four 5-mm trocars were placed on the left side in the left flank position, while two 5-mm trocars were added for the right side in the right flank position (totally 6 trocars). On the left, the adrenal mass, located above the left kidney, was excised with totally preservation of adrenal tissue (Fig. 2). Bipolar cautery was employed for adrenal hemostasis. On the right, the adrenal mass was positioned under the liver, which was retracted upward for access. The mass was carefully separated from the upper pole of the kidney and surrounding tissues. A 4-cm mass was removed, again with almost no adrenal tissue remaining on the resected specimen (Fig. 2).

**2):**
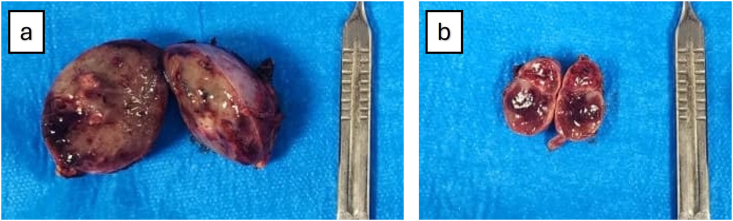
Bilateral adrenal masses: (a) right side and (b) left side.

Our approach, utilizing tumorectomy instead of adrenalectomy, avoided exposing the renal and adrenal veins on the left side as well as the IVC and adrenal vein on the right side, making the surgery faster and less invasive. This technique offers a significant advantage in bilateral cases by preserving adrenal glands, thereby eliminating the need for lifelong corticosteroid and mineralocorticoid hormone replacement therapy. The specimens were extracted through the enlarged umbilical port.

The procedure lasted 150 minutes and was completed without requiring a blood transfusion. No intraoperative hypertension or arrhythmias occurred during the laparoscopic surgery.

Postoperatively, the patient experienced a mild blood pressure drop, managed with normal saline and hydrocortisone (50 mg every 8 hours), which was discontinued the following day. The patient showed no significant hemoglobin decrease during hospitalization, and the surgical drain was removed on the third day due to minimal output. The patient was discharged in stable condition after three days. Pathological evaluation confirmed the diagnosis of bilateral pheochromocytoma.

**Follow-up:** At the six-month follow-up, the patient exhibited no symptoms such as high blood pressure and showed no signs of adrenal insufficiency, eliminating the need for corticosteroid or mineralocorticoid replacement therapy. Repeat 24-h urine tests indicated normal levels of cortisol, metanephrine and normetanephrine (Table 1), and a follow-up MRI confirmed the absence of residual tumor (Fig. 1).

**Table 1:** Laboratory tests before and after surgery.

	Before surgery	After surgery (6 months)	Normal range
24-h urine metanephrine (μg/24 h)	513	180	Up to 350
24-h urine normetanephrine (μg/24 h)	2671	386	Up to 600
serum Cortisol 8AM(micg/dl)	12	12.9	6–18
serum aldosterone(pg/dl)	178	200	20–220
serum renin (pg/dl)	33	29	5.41–34.5

## 3 Discussion

Laparoscopic adrenalectomy was first introduced nearly 30 years ago, followed by partial adrenalectomy over 20 years ago. Both techniques are now well-established in adults. Over the last decade, minimally invasive approaches to adrenalectomy have gained wider acceptance for treating children with pheochromocytoma.^9^^,^^10^ Managing pheochromocytoma in pediatric patients poses unique challenges. While total adrenalectomy remains the traditional standard for bilateral tumors, it carries the risk of adrenal insufficiency, requiring lifelong hormone replacement therapy.^3^^,^^7^^,^^11^^,^^12^ This study, alongside the existing literature, emphasizes the feasibility and benefits of laparoscopic bilateral adrenal-sparing surgery in preserving adrenocortical function, thereby avoiding glucocorticoid and mineralocorticoid replacement.

The main advantage of laparoscopic bilateral adrenal-sparing surgery lies in its ability to preserve adrenocortical function. Particularly in hereditary pheochromocytoma, such as in Von Hippel-Lindau (VHL) disease, partial adrenalectomy enables tumor removal while maintaining sufficient healthy adrenal tissue to ensure normal hormone production.^13^ This approach is especially critical in pediatric patients, for whom lifelong hormone therapy poses significant challenges for growth, development, and quality of life. Using clipless techniques minimizes trauma to the remaining adrenal tissue, further enhancing functional outcomes. Previous studies highlight the effectiveness of clipless laparoscopic techniques when performed by experienced surgeons.^10^^,^^14^ Accordingly, in this case, we utilized a clipless adrenal-sparing approach to optimize adrenal gland function, distinguishing it from other reported cases.

One of the most notable benefits of adrenal-sparing surgery is the elimination of lifelong hormone replacement therapy. Studies consistently show that patients undergoing partial adrenalectomy avoid glucocorticoid or mineralocorticoid supplementation, provided adequate adrenal tissue is preserved.^15^ This reduces the burden of daily medication while mitigating risks linked to long-term steroid use, such as osteoporosis, weight gain, and metabolic disturbances.

Laparoscopic adrenal-sparing surgery also reduces surgical morbidity compared to open procedures. Its minimally invasive nature results in less postoperative pain, shorter hospital stays, and faster recovery.^9^^,^^13^ Pediatric patients especially benefit, recovering more quickly and resuming normal activities sooner. In this case, we performed bilateral tumorectomy with minimal dissection of surrounding adrenal tissue and blood vessels, preserving normal tissue and adrenal function. Preservation of adrenal function has profound implications for quality of life. By avoiding hormone replacement therapy and minimizing surgical morbidity, patients experience fewer complications and maintain normal physiological states. This is particularly significant in pediatric patients, who face unique challenges related to growth, development, and psychosocial well-being.

While the short-term benefits of laparoscopic adrenal-sparing surgery are well-established, long-term outcomes are equally promising. Studies indicate low tumor recurrence rates in patients undergoing partial adrenalectomy, provided regular follow-ups are maintained.^15^ This underscores the need for a multidisciplinary approach to managing hereditary pheochromocytoma, involving collaboration between surgeons, endocrinologists, and oncologists.

Although our case demonstrated excellent short-term outcomes, long-term surveillance remains essential to monitor for recurrence. Recurrence rates of 10–15 % over 10 years have been reported in adrenal-sparing cohorts.^1^ Additionally, standardized postoperative steroid tapering protocols, tailored to remnant adrenal volume, warrant further study.

## 4 Conclusion

Laparoscopic bilateral synchronous adrenal-sparing surgery has emerged as a crucial advancement in treating pediatric pheochromocytoma. This case report highlights the successful application of this technique in an 11-year-old boy with bilateral pheochromocytoma. The procedure, performed without the use of clips, proved to be a viable alternative to total adrenalectomy by effectively removing tumors while preserving adrenal function. Administering full alpha blockade with phenoxybenzamine is strongly recommended to avoid intraoperative and postoperative complications.

## CRediT authorship contribution statement

**Nasser Simforoosh:** Conceptualization, Data curation, Formal analysis, Methodology, Project administration, Supervision, Writing – review & editing, Investigation. **Javad Nikbakht:** Methodology, Writing – original draft. **Ahmad Eghbali:** Conceptualization, Investigation, Methodology. **Nooshin Ahmadi:** Conceptualization, Data curation, Formal analysis, Methodology. **Mohammad Sajjad Zabihi:** Writing – original draft. **Saeedeh Sarhadi:** Writing – original draft. **Mehdi Dadpour:** Conceptualization, Data curation, Formal analysis, Supervision, Writing – original draft, Writing – review & editing.

## Funding statement

No fund was used in this project.

## Declaration of competing interest

All authors declare that they have no conflict of interests.
